# Mitochondrial dysfunction contributes to Rapamycin-induced apoptosis of Human Glioblastoma Cells - A synergistic effect with Temozolomide

**DOI:** 10.7150/ijms.40159

**Published:** 2020-10-16

**Authors:** Mary A Zimmerman, Samantha Wilkison, Qi Qi, Guisheng Chen, P. Andy Li

**Affiliations:** 1 Department of Pharmaceutical Sciences, Biomanufacturing Research Institute Biotechnology Enterprise (BRITE), North Carolina Central University, 1801 Fayetteville St, Durham, NC, 27707, USA.; 2 Department of Biology, University of Wisconsin-La Crosse, 1725 State St, La Crosse, WI, 54601, USA.; 3 Department of Pharmacology and Cancer Biology, Duke University, Durham, NC, 27708, USA.; 4 Department of Neurology, Neuroscience Center, General Hospital of Ningxia Medical University, and Key Laboratory of Craniocerebral Diseases of Ningxia Hui Autonomous Region, Yinchuan 750004, China.

**Keywords:** Rapamycin, glioblastoma, mTOR signaling, mitochondrial dynamics, mitochondrial dysfunction, Temozolomide

## Abstract

Mammalian target of rapamycin (mTOR) is upregulated in a high percentage of glioblastomas. While a well-known mTOR inhibitor, rapamycin, has been shown to reduce glioblastoma survival, the role of mitochondria in achieving this therapeutic effect is less well known. Here, we examined mitochondrial dysfunction mechanisms that occur with the suppression of mTOR signaling. We found that, along with increased apoptosis, and a reduction in transformative potential, rapamycin treatment significantly affected mitochondrial health. Specifically, increased production of reactive oxygen species (ROS), depolarization of the mitochondrial membrane potential (MMP), and altered mitochondrial dynamics were observed. Furthermore, we verified the therapeutic potential of rapamycin-induced mitochondrial dysfunction through co-treatment with temzolomide (TMZ), the current standard of care for glioblastoma. Together these results demonstrate that the mitochondria remain a promising target for therapeutic intervention against human glioblastoma and that TMZ and rapamycin have a synergistic effect in suppressing glioblastoma viability, enhancing ROS production, and depolarizing MMP.

## Introduction

Glioblastoma multiforme (GBM) is a highly aggressive tumor typically arising in the brain or spinal cord. Even with conventional therapies, such as surgery, radiation, or chemotherapy, the mean survival rate remains around 15 months 1. Temozolomide (TMZ) is one of the only few Food and Drug Administration-approved drugs for glioblastoma therapy. Cytotoxicity from TMZ treatment, however, is often short-lived as highly aggressive, surviving tumor cells repopulate leading to TMZ-resistant GBM recurrence in patients 2, 3. Searching for new compounds that could decrease the TMZ-resistance is in high demand. Given the lack of effective secondary treatment options, new therapeutic strategies targeting resistance mechanisms, as well as the signaling pathways most affected in glioblastoma cells, are greatly needed. One such pathway, shown to be genetically altered in 88% of gliomas, is the receptor tyrosine kinase (RTK) pathway 4. This pathway, triggered through engagement of the epidermal growth factor receptor (EGFR), includes the phosphoinositide 3-kinase (PI3K)/protein kinase B (AKT)/mTOR signaling pathway. Hyperactivation of mTOR is often seen in glioblastoma and its high expression has been correlated to a poorer prognosis and a decrease in survival 5, 6.

mTOR is a serine-threonine kinase that acts in a multifunctional pathway with complex roles in metabolism, cell proliferation, RNA translation, apoptosis, autophagy, and angiogenesis 7,8. The therapeutic potential of rapamycin has been studied extensively 9-15. Rapamycin is most recognized for its ability to inhibit mTOR signaling. Of particular interest to our study is that reducing mTOR signaling via rapamycin has been documented to decrease glioblastoma viability 16-18. However, one of the road blocks to using rapamycin as an effective glioblastoma therapy is that, like with TMZ therapy, resistance often develops 19, 20. The mechanism of resistance is suggested to involve increased pro-survival AKT signaling through mTORC2 that is activated as a counterbalance to reduced mTOR levels via rapamycin's inhibition of mTORC1 19, 21. Since AKT can then stimulate mTOR transcription, this may essentially result in an increase in mTOR, rather than the desired decrease, and represents one possibility for rapamycin's failure in clinical trials treating GBM.

Despite these challenges, rapamycin can elicit intracellular changes outside of the PI3K/mTOR/AKT axis, most notably by evoking changes in autophagy, which can greatly influence a cell's fate 22. In a previous study, we showed that rapamycin could promote cell survival during CoCl_2_-simulated hypoxia in HT22 neuronal cells, independent of changes in mTOR signaling, and primarily through improving mitochondrial health. The mitochondria play a role in mediating TMZ resistance by changing its respiration, ROS production and signal transduction 23-25. Since rapamycin has been reported to induce apoptosis in glioblastoma, we wanted to determine the rapamycin-induced effects on mitochondria, in addition to decreasing mTOR signaling, as a means of searching for new mitochondria-targeted therapies to treat GBM.

Mitochondrial function is highly dependent on oxygen consumption and can be severely disrupted during instances of hypoxia. Hypoxia, a state of reduced oxygen, has a somewhat complicated relationship with cancer. Hypoxia can promote cancer development by increasing ROS production. An overabundance of ROS can stimulate cancer-inducing mutations in the cell's DNA through oxidation reactions 26. Many cancers are known to produce higher than normal levels of ROS, but do not suffer losses in viability from it. Often as cancerous cells grow faster than angiogenesis can keep pace providing the oxygen-carrying blood supply, pockets of cells will adapt to the low oxygen availability and thrive under these conditions 27. Glioblastoma is known for its particularly hypoxic environment and its ability to adapt and thrive under this condition. Therefore, targeting the mitochondria for disruption within this environment may serve to work against these adaptive mechanisms and re-trigger glioblastoma cells to undergo apoptosis.

Based on the metabolic and function needs, the mitochondria maintain a balance between fusion and fission. In general, the fusing of two mitochondria into one (fusion) is stimulated by increased energy demands and stress. An increase in fusion will yield elongated and highly interconnected mitochondria allowing resources, such as lipid contents, to be pooled. Key proteins (GTPases) mediating fusion are Mitofusins 1 and 2 (MFN1/2) which work to fuse the outer mitochondrial membranes of two adjacent mitochondria, and optic atrophy 1 (OPA1) which mediates fusion of the inner mitochondrial membranes 28. On the other side of the scale, the process of mitochondrial division (fission) generates new organelles and facilitates quality control. Dynamin-related protein 1 (DRP1) is a key protein mediating the fission process. With the help of a second protein, mitochondrial fission protein 1 (FIS1), anchoring DRP1 to the mitochondria, DRP1 wraps around and constricts the mitochondrion until it separates forming two distinct organelles 28. This mechanism allows an injured mitochondrion to cordon off damaged sections. Once divided, the damaged part can be removed from the cell through autophagic processes. In this way, the undamaged section can continue to function rather than necessitating complete loss of an injured mitochondrion. This also prevents the accumulation of damage-induced DNA mutations. Disruption of mitochondrial dynamics has been observed early during apoptosis and cancer development 29,30.

Since there are very few effective treatments for glioblastoma, this study provides proof-of-concept for targeting the mitochondria to induce cell death of glioblastoma. In this present study our objectives were to 1) ascertain the cytotoxic effect and hence the therapeutic potential of rapamycin treatment on U87-MG human glioblastoma cells; 2) assess the disruption of mitochondrial processes (mitochondrial dysfunction) during rapamycin treatment; and 3) examine the synergistic effect of rapamycin and TMZ combination in glioblastoma cells. Specifically, we determined the effects on mitochondrial membrane potential, generation of ROS production, and changes in expression of key proteins mediating mitochondrial dynamics.

## Materials and Methods

### Cell culture

U87-MG (ATCC^®^ HTB-14™) is a human glioblastoma cell line of central nervous system origin 31 obtained from American Type Culture Collection (ATCC, Manassas, VA). ATCC characterizes their cell lines by morphology, immunology, DNA fingerprint, and cytogenetics. U87-MG cells were cultured in Dulbecco's Modified Eagles Medium (DMEM)-High Glucose (GE Healthcare Life Sciences, UT) supplemented with 10% Fetal Bovine Serum, L-glutamine (2 mM), and antibiotics penicillin G and streptomycin (200 units/mL) (Thermo Fisher Scientific, UT). Cells were maintained in a 5% CO_2_ incubator at 37 ºC, and 90-95% humidity.

### Drug treatment

Rapamycin dose response was tested in concentrations of 5, 10, 20, 30, 40, 50 and 60 μM and TMZ in concentrations of 0.5, 1, 2, 3, and 4 mM. For combination therapy of rapamycin and TMZ, rapamycin 20 and 30 μM and TMZ 1 mM were selected. Cells were incubated with drugs for 4 or 24 h before sampling.

### Measurement of cell viability via resazurin assay

Cell viability was measured using a water-soluble, indicator dye, resazurin (7-Hydroxy-3H-phenoxazin-3-one 10-oxide), essentially as previously described 32. Resazurin sodium salt (Acros Organics, NJ) was dissolved in media at a final concentration of 0.1 mg/mL and 10% volume. This cell-permeable dye is internalized by cultured cells. Actively growing cells metabolize resazurin into a fluorescent form, resorufin. Resultant fluorescence was measured using a PHERAstar Microplate Reader (BMG Labtech, NC) with a 540-20/590-20 filter. The viability of control cells was arbitrarily set to 100 % and the relative fluorescence intensities of experimental groups were converted to relative percentages using the formula: (Relative Fluorescence Intensity of Experimental/Average Relative Fluorescence Intensity of Control) × 100 = percentage of viable cells.

### Western blotting

Cells for Western blot analysis were lysed to obtain either cytosolic and mitochondrial, or cytosolic and nuclear protein fractions. To obtain the cytosolic and mitochondrial protein fractions, cells were lysed in cytosol extraction buffer (250 mM sucrose, 70 mM KCl, 137 mM NaCl, 4.3 mM Na_2_HPO_4_, 1.4 mM KH_2_PO_4_ pH 7.2, and 200 µg/mL digitonin) plus phosphatase and proteinase inhibitors (Pierce and Thermo Fisher Scientific) on ice for 5 min. Cells were centrifuged at 1000 ×*g* for 5 min at 4 °C, before reserving the supernatant as the cytosolic fraction. The cytosolic fraction was further cleared of debris by centrifugation at 20,000 ×*g* for 10 min at 4 °C. Meanwhile, the mitochondrial fractions were obtained by incubating the pellet from the first, low-speed centrifugation in two volumes of mitochondrial lysis buffer (50 mM Tris-HCl pH 7.4, 150 mM NaCl, 2 mM EDTA, 2 mM EGTA, 0.2 % (v/v) Triton X-100, and 0.3 % NP-40) plus the above inhibitors. Nuclear protein fractions were obtained essentially as previously described 33.

Protein concentrations were measured using standard Bradford Assays (Bio-Rad Laboratories, Hercules, CA). Protein lysates (20 µg per well) were separated using 4-12% Bis-Tris NuPAGE gels except in the cases of mTOR/phosho-mTOR detection where 3-8 % Tris-Acetate NuPAGE gels were used according to the manufacturer's instructions (Invitrogen, Carlsbad, CA). The Bio-Rad Mini Trans-Blot system was used to transfer the separated proteins to polyvinylidene fluoride membranes. After transfer, membranes were blocked in a 1:1 solution of Li-COR Odyssey Blocking buffer (Li-COR, Inc., Lincoln, NE) and PBS. Membranes were then probed using the indicated primary antibodies, all obtained from Cell Signaling Technology (Danvers, MA), at 1:1000 dilutions, except in the case of cytosolic loading control β-actin, which was diluted 1:5000. IRDye 680LT goat anti-rabbit and IRDye 800CW goat anti-mouse secondary antibodies from Li-COR, Inc (Lincoln, NE) were used at 1:10,000 dilutions for visualization using the Li-COR Odyssey Classic Imaging System scanner. Images obtained using this scanner were analyzed with the Li-COR Image Studio Software version 5.2.5. Fluorescent signals were normalized to loading controls β-actin, COX IV, or Histone 3, for cytosolic, mitochondrial, or nuclear protein fractions respectively. Average relative protein expressions of experimental treatment groups were determined by comparison to average expression of the control.

### Measurement of rapamycin-induced apoptosis

After treatment with 0, 20, 40 or 60 µM rapamycin for 24 hours, U87-MG cells were stained with apoptotic indicators, Fluorescein isothiocyanate-conjugated Annexin V (AV) and Propidium Iodide (PI), according to the manufacturer's instructions (BD Biosciences, San Diego, CA). Cell staining was analyzed using a CytoFlex flow cytometer (Beckman Coulter, Indianapolis, IN). The average percent of double positive-stained cells in each rapamycin treatment group was quantified and compared to untreated controls.

### Assay for measurement of reactive oxygen species production

U87-MG cells were either untreated or treated for 24 hours with 60 µM Rapamycin in 96 well plates with cells at around 70% confluence. Five μM dihydroethidium (DHE) in DMEM was added during the last 30 min of treatment time with incubation continuing at 37 °C. DHE is a cell permeable dye that becomes oxidized into a fluorescent compound, 2-hydroxyethidium, when the ROS indicator, superoxide, is produced in cells. Increased fluorescence, therefore, corresponds to increased ROS production. At the end of the 24 h treatment time, media was removed, and cells were washed twice with PBS. A final volume of 100 µL PBS was added to each well prior to measuring fluorescence using a PHERAstar Microplate Reader with a 590-50/675-50 filter. Background fluorescence was subtracted using additional treatment sets without DHE. To compensate for fluorescence signal changes caused by cell death, resazurin cell viability assays, as described above, were performed in parallel using the same rapamycin treatments used to measure ROS production. Fluorescence measurements were normalized against cell viability to calculate the relative fluorescence values of control versus rapamycin-treated cells in which an increase in fluorescence is indicative of an increase in ROS production.

### Assay for measurement of the mitochondrial membrane potential

Measurement of MMP was essentially as previously described 32. U87-MG cells were cultured in 96 well plates to around 70% confluence. Cells were then either untreated or treated with 40 μM Rapamycin for 24 hours at 37 °C with 500 nM tetramethylrhodamine, methyl ester, perchlorate (TMRM) being added during the final 30 minutes of treatment time. Fluorescence was measured using a PHERAstar Microplate Reader (BMG Labtech, Durham, NC) using a 590-50/675-50 filter.

### Soft agar colony formation assay

1.5 mL per well of a base layer of DMEM media containing additives described above for culture of U87-MG cells, and 0.6 % agarose, was added to 6-well plates and allowed to solidify. Once solid, a 2 mL top layer containing the same media components as the base, but with a decrease to 0.3 % agarose and 1 × 10^4^ U87-MG suspended cells, was added over the base layer of each well. U87-MG cells were previously treated for 24 hours with 0 or 20 µM rapamycin before being seeded in this semi-solid, soft agar layer. Cells were fed 0.5 mL fresh media once or twice a week as needed and incubated for 14 days at 37 °C to allow the formation of colonies. Cell colonies were then stained with a 0.005% crystal violet solution overnight. Stained wells were imaged, and the number of colonies were counted using ImageJ software version 1.49 34.

### Statistical Analysis

Each experiment was repeated at least three times. Data is presented as either mean values ± standard deviation (SD), or as a percentage of the control. Statistical analyses were carried out using one-way ANOVA followed by Bonferroni's test for multiple comparisons, with *p*-values <0.05 considered statistically significant.

## Results

### Treatment with rapamycin leads to reduced cell viability and induction of apoptosis

**Figure 1:**
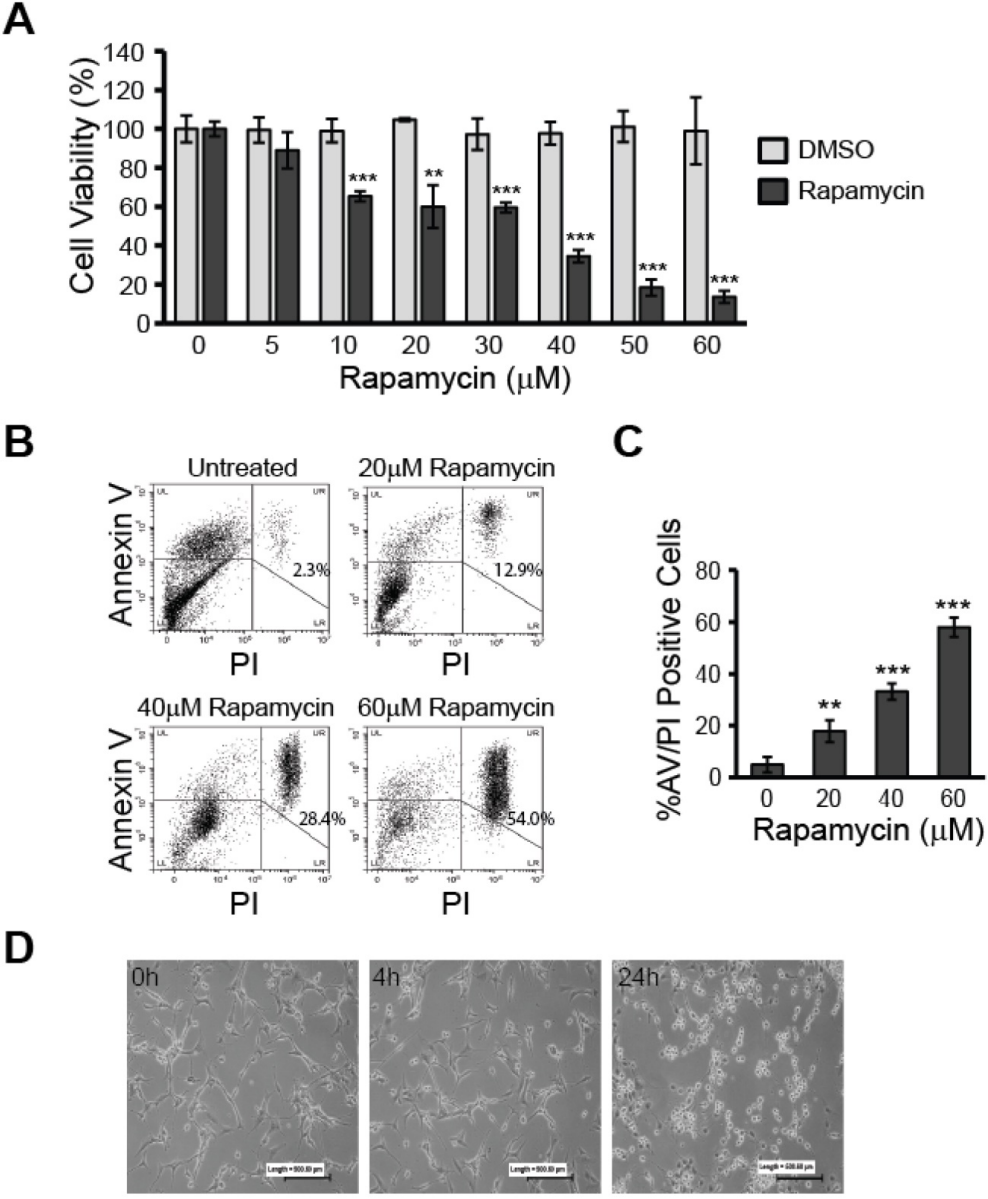
** Rapamycin treatment reduces viability and induces apoptosis in U87-MG glioblastoma cells. A)** Rapamycin decreases glioblastoma viability in a dose-dependent manner. U87-MG cells were treated at 70 % confluence in 96-well plates with the indicated concentrations of rapamycin (0-60 µM) for 24 h followed by assessment of cell viability using the resazurin viability assay. ** *p<*0.01 and *** *p<*0.001 vs control. **B)** Rapamycin induces apoptosis in a dose-dependent manner. Cells were treated with 0, 20, 40, or 60 µM rapamycin for 24 h before collection and staining with AV and PI. Cells were then analyzed by flow cytometry for apoptosis. Representative dot plots are shown for each treatment condition with the percentage of cells staining positive for both AV and PI indicated in the upper right quadrant. LL = lower left quadrant; cells negative for staining, LR = lower right quadrant; cells PI-positive only, UL = upper left quadrant; cells AV-positive only, UR = upper right quadrant; double-positive AV/PI stained cells.** C)** Average percent of apoptosis induced by rapamycin treatment. Apoptotic cell death was quantified by averaging the percent of AV/PI-positive cells in each treatment group and comparing to control. ** *p<*0.01 and *** *p<*0.001 *vs*. control. **D)** Morphological changes associated with rapamycin treatment. Microphotographs of U87-MG cells treated for 0, 4, or 24 h with 40 µM rapamycin. Cells were imaged under 10X magnification using a standard light microscope.

Before exploring rapamycin-induced changes to relevant molecular pathways and effects on mitochondria, we first verified whether rapamycin exhibited a therapeutic effect in U87-MG human glioblastoma cells as having been previously reported. Using resazurin viability assays, we assessed cell viability over a range of rapamycin concentrations from 5-60 µM and compared the effects against its vehicle, dimethyl sulfoxide (DMSO). As can be seen in Figure 1A, rapamycin decreased U87-MG viability in a dose-dependent manner with cells becoming significantly affected by concentrations of 10 µM and above (*p<*0.001).

To verify that cells treated with rapamycin undergo apoptosis, rather than only experiencing slowed metabolism, we used flow cytometry analysis to measure the percent of AV/PI-positive cells after 24 h of treatment with 0, 20, 40, or 60 µM rapamycin (Figure 1B and C). The percent of AV/PI-positive cells increased by roughly 12.9% with 20 µM rapamycin treatment (*p<*0.01). Doubling the rapamycin dose also led to a doubling of the percent of AV/PI-positive cells with 40 µM rapamycin causing 28.4% of cells to express the apoptosis markers (*p<*0.001). Our highest dose of 60 µM rapamycin resulted in an average of nearly 54.0% of cells becoming AV/PI positive (*p<*0.001).

Based on these results, we chose 40 µM rapamycin to use in our subsequent experiments as this concentration was effective at inducing significant apoptosis and reduced cell viability by over 60 %. The effect of 40 µM rapamycin treatment on gross cell morphology could also be seen in cultured cells at 4 and 24 h time points. Observation under a light microscope at 10X magnification showed U87-MG cells beginning to round up and detach from the culture plate at 4 h of treatment and extensive cell death was apparent at the 24 h time point (Figure 1D).

### Rapamycin characteristically targets the mTOR protein and affects both upstream and downstream signaling

**Figure 2:**
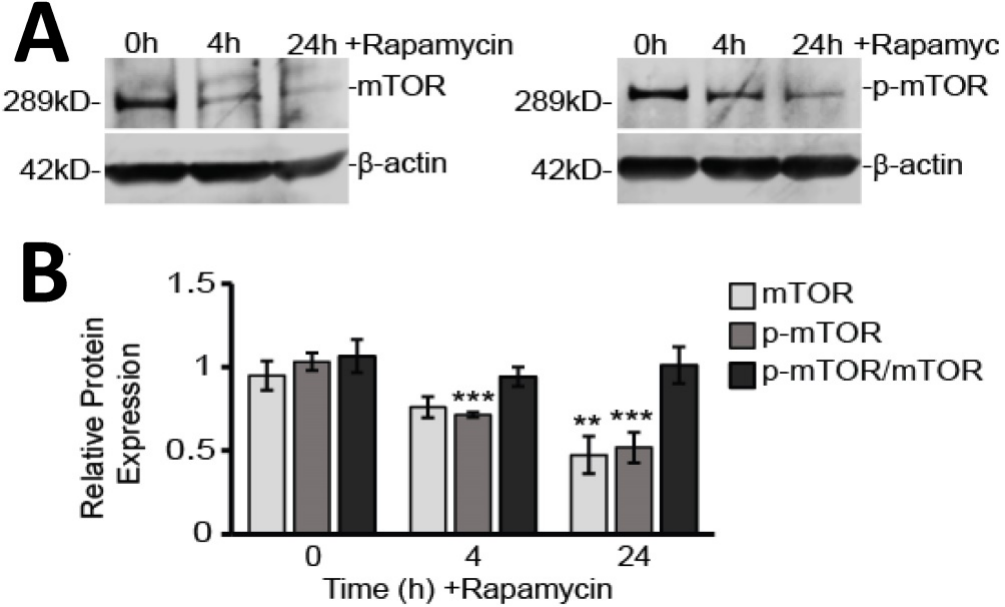
** Rapamycin treatment inhibits mTOR in glioblastoma cells. A)** Representative Western blot images for mTOR and p-mTOR cytosolic protein expression with β-actin as a loading control. Results are representative of at least three Western blots.** B)** Western blot analysis showing average cytosolic protein expression, relative to 0 h control, of mTOR and p-mTOR after normalizing fluorescent signals to β-actin. Also shown is the ratio of p-mTOR to mTOR. Cytosolic protein fractions were used from cells treated for 0, 4, and 24 h with 40 µM Rapamycin. ** *p<*0.01 and *** *p<*0.001 *vs*. 0 h.

Rapamycin is a well-known inhibitor of mTOR and so it was not surprising we found that both unphosphorylated and phosphorylated mTOR (p-mTOR) protein levels were reduced by rapamycin treatment (Figure 2A and B). Western blot analysis showed p-mTOR decreased by 30% after 4 h (*p<*0.001) and 60% after 24 h (*p<*0.001) of rapamycin treatment. Unphosphorylated mTOR protein slowly declined by only 20% after 4 h (p>0.05) and by 40% after 24 h (*p<*0.01). As both mTOR and p-mTOR expression declined together over time, the overall ratio of p-mTOR to mTOR was unchanged.

**Figure 3:**
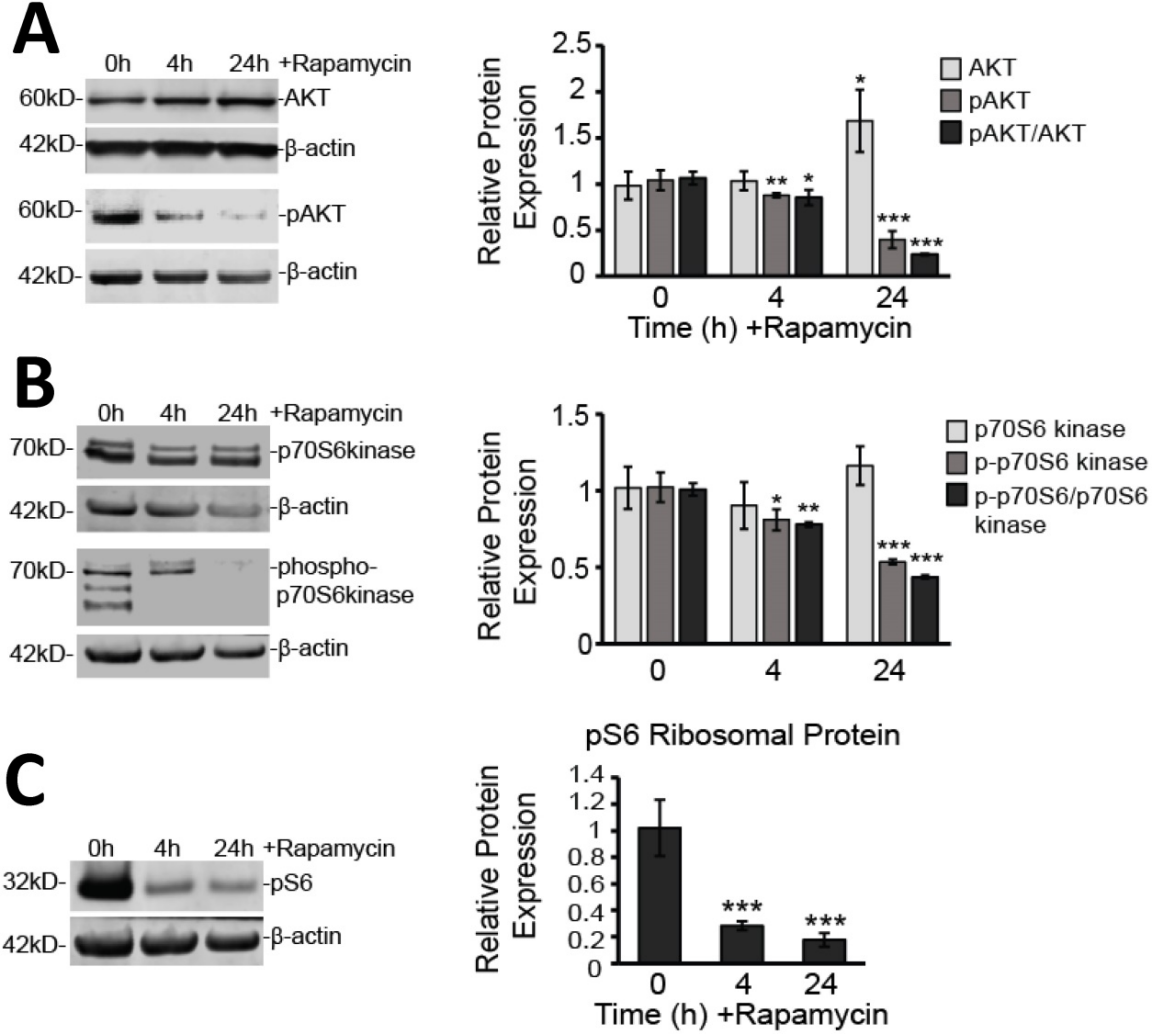
** Rapamycin inhibits mTOR signaling both upstream and downstream of mTOR. A)** Rapamycin inhibits upstream effector of mTOR expression, AKT. Cytosolic protein fractions were used from cells treated for 0, 4, and 24 h with 40 µM Rapamycin. Left: Western blot images for AKT and pAKT representative of at least three Western blots. β-actin is shown as a loading control. Right: Western blot analysis showing average protein expression, relative to 0 h control, of AKT and pAKT after normalizing fluorescent signals to β-actin. Also shown is the ratio of pAKT to AKT. **B)** Rapamycin inhibits downstream target of mTOR, p70 S6K. Cells were treated as in A. Left: Western blot images for p70 S6K and p-p70 S6K representative of at least three western blots. β-actin is shown as a loading control. Right: Western blot analysis showing average protein expression, relative to 0 h control, of p70 S6K and p-p70 S6K after normalizing fluorescent signals to β-actin. Also shown is the ratio of p-p70 S6K to p70 S6 K. **C)** Rapamycin inhibits downstream target of mTOR, pS6. Cells were treated as in A. Left: Western blot images for pS6 protein representative of at least three Western blots. β-actin is shown as a loading control Right: Western blot analysis showing average protein expression, relative to 0h control, of pS6 ribosomal protein after normalizing fluorescent signals to β-actin. * *p<*0.05, ** *p<*0.01 and *** *p<*0.001 *vs*. 0 h.

We also verified that downstream mTOR signaling pathways were significantly affected. The mTORC1 complex has been shown to regulate protein translation through activation of p70 S6 kinase (p70 S6K) and to enhance RNA translation via S6 ribosomal protein (pS6) 35. While expression of the p70 S6K protein itself was not affected by rapamycin treatment, its phosphorylated form (p-p70 S6K), as well as the overall ratio of p-p70 S6K to p70 S6K, were significantly reduced (Figure 3A). The p-p70 S6K/p70 S6K ratio decreased by 22% at 4 h (*p<*0.01) and 57% at 24 h treatment suggesting impaired phosphorylation (*p<*0.001). Even further downstream, pS6, which is considered a reliable marker for signaling through the mTORC1 complex, was also reduced by 72% at 4 h and 83% at 24 h rapamycin treatment (*p<*0.001) (Figure 3B).

In addition to downstream signaling, we examined AKT signaling upstream of mTOR (Figure 3C). We found that AKT protein expression was elevated after 24 h of rapamycin treatment by 71% (*p<*0.05). However, phosphorylated AKT (pAKT) showed significant decreases in expression at 4 h (17%; *p<*0.01) and at 24 h (61%; *p<*0.001). The overall ratio of pAKT/AKT decreased by 17 % at 4 h (*p<*0.01) and 77% at 24 h (*p<*0.001).

### Glioblastoma cell death is promoted through depolarization of the mitochondrial membrane and increased production of ROS

**Figure 4:**
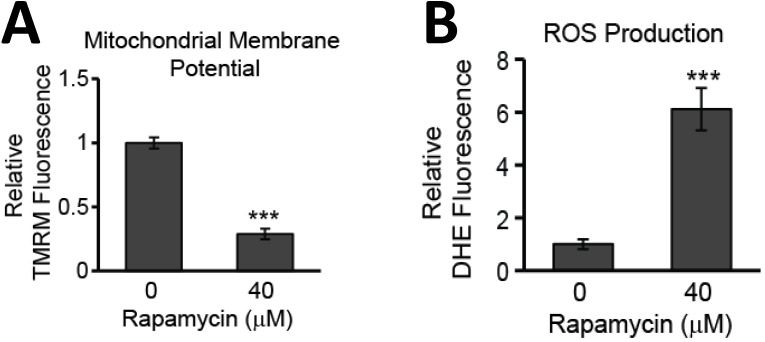
** Rapamycin treatment destabilizes the MMP and increases the production of ROS in glioblastoma cells. A)** Rapamycin treatment destabilizes the mitochondrial membrane. U87-MG cells were untreated or treated with 40 µM rapamycin for 24 h with 500 nM TMRM dye being added during the final 30 min of treatment. **B)** Rapamycin increases ROS production. Cells were treated as in A except 5 µM DHE dye was added instead of TMRM. In both A and B, fluorescence was measured using a PHERAstar Microplate Reader with a 590-50/675-50 filter. Relative fluorescence intensities were obtained by subtracting background signal from treated cells without TMRM/DHE and normalizing to the percent of viable cells. *** *p<*0.001 *vs.* 0 h.

While evidence of rapamycin modifying mTOR signaling pathways is abundant in the literature, less well documented are the effects of rapamycin on mitochondrial health. The mitochondrial membrane potential is often used as a measure of mitochondrial integrity. Alterations to the inner membrane, such as depolarization or hyperpolarization, can lead to permeabilization of the outer mitochondrial membrane (MOMP). MOMP then leads to release of a slew of pro-apoptotic proteins normally kept sequestered between these two mitochondrial membranes. We have previously observed rapamycin altering the mitochondrial membrane in mouse hippocampal cells 22 and believed this response could be contributing to the rapamycin-induced toxicity in U87-MG cells. Using a fluorogenic dye, TMRM, as described in the Materials and Methods, we found that after 24 h of rapamycin treatment, the mitochondrial membrane potential in U87-MG cells undergoes significant depolarization as evidenced by a drop in fluorescence of 71% (*p<*0.001) (Figure 4A).

In addition to promoting mitochondrial-mediated apoptosis (the intrinsic cell death pathway), depolarization of the mitochondrial membrane can lead to increases in toxic ROS production as oxidative phosphorylation along the inner mitochondrial membrane becomes compromised. The quick reaction rate of newly generated ROS makes the mitochondria particularly sensitive to ROS-induced damage causing further malfunction of mitochondrial processes. We too observed that U87-MG cells experiencing mitochondrial membrane depolarization also experienced a roughly 5-fold increase in ROS production (*p<*0.001) (Figure 4B).

### Disruption of mitochondrial dynamics occurs during rapamycin-induced cell death

**Figure 5:**
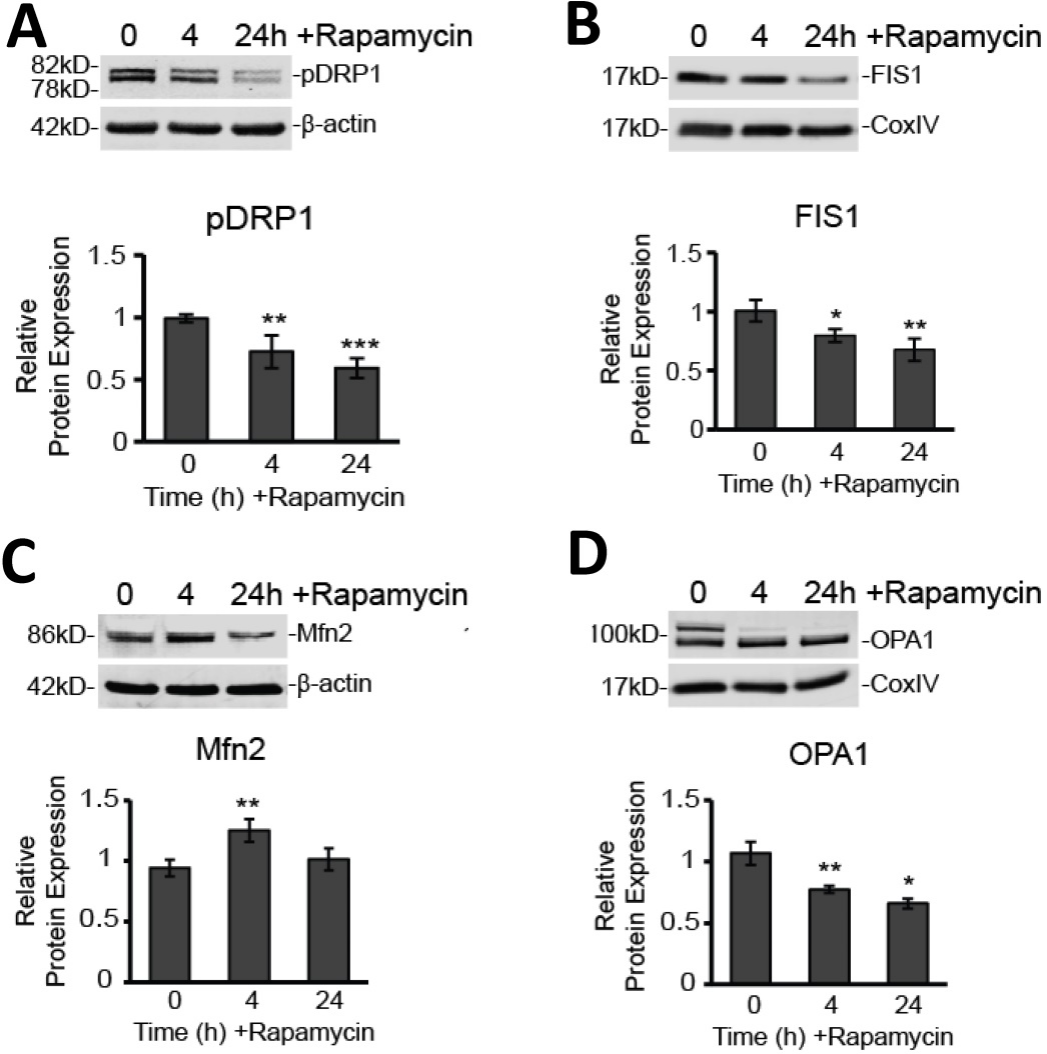
** Rapamycin treatment alters expression of fission and fusion proteins. A)** Rapamycin reduces protein expression of pDRP1. Western blot analysis showing average total protein expression for pDRP1 relative to 0 h control and a representative blot image after treatment with 40 µM rapamycin for 4, and 24 h. Protein content was normalized to β-actin fluorescent signal. **B)** Rapamycin reduces protein expression of FIS1. Western blot analysis showing average mitochondrial protein expression relative to 0 h control of FIS1 after treatment with 40 µM rapamycin for 4, and 24 h. Protein content was normalized to CoxIV fluorescent signal. **C)** Rapamycin briefly increases protein expression of phosphorylated MFN2. Cells were treated as in 5A for Western blot analysis of MFN2. Protein content was normalized to β-actin fluorescent signal.** D)** Rapamycin reduces protein expression of OPA1. Cells were treated as in 5B and Western blot analysis for average mitochondrial protein expression of OPA1. Protein content was normalized to Cox IV fluorescent signal. * *p<*0.05, ** *p<*0.01 and ***p<0.001 *vs.* 0 h. All results are from at least three separate experiments.

Since we believe such catastrophic targeting of the mitochondria via alteration of the membrane potential and production of ROS may prove key to glioblastoma therapy, we further explored how mitochondrial dynamics are affected by rapamycin treatment. Specifically, we used Western blot analysis to observe protein expression changes in key proteins mediating mitochondrial fission and fusion. We found that, after rapamycin treatment, both fission proteins, DRP1 and FIS1, were significantly reduced (Figure 5A and B). Phosphorylated DRP1 (pDRP1) was decreased by 27% at 4 h treatment (*p<*0.01) and by 40% at 24 h treatment (*p<*0.001). Similarly, FIS1 experienced a 20 % decrease at 4 h (*p<*0.05) and a 32% decrease at 24 h (*p<*0.01).

On the other hand, Western blot analysis showed that the outer mitochondrial membrane fusion protein, MFN2, was upregulated by nearly 30% (*p<*0.01) at 4 h rapamycin treatment, but then returned to baseline at 24 h (Figure 5C). In contrast, the protein mediating inner mitochondrial membrane fusion, OPA1, was significantly downregulated at 4 h (28%; *p<*0.01) and 24 h (38%; *p<*0.05) of rapamycin treatment (Figure 5D).

### The transformative potential of glioblastoma is reduced after rapamycin treatment

**Figure 6:**
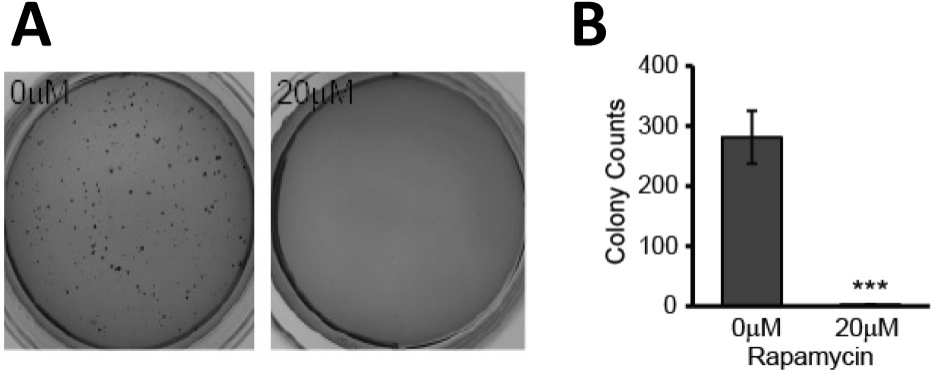
** Rapamycin treatment causes loss in glioblastoma transformative potential. A)** Effects of rapamycin treatment on colony formation in soft agar. Representative photos of soft agar assay wells showing formation of colonies in each treatment group. Cells were treated for 24 h with 0 or 20 µM rapamycin prior to being seeded in semi-solid soft agar. Once seeded, cells were incubated for 2 weeks at 37 °C prior to staining with crystal violet for visualization and quantification. **B)** Average number of colonies formed is reduced with rapamycin treatment. Graph showing the average number of colonies formed in each group after being treated as in 6A. Results are representative of three separate experiments. *** *p<*0.001 vs control.

Our results indicated that the mitochondria are negatively affected and may play a key role in mediating rapamycin-induced apoptosis. In addition to inducing apoptosis of existing cells, treatment with rapamycin effectively hindered the clonogenic ability of U87-MG cells. This reduction in transformative potential was evidenced by a reduction in anchorage-independent colony formation in semi-solid agar. Cells were treated with and without rapamycin for 24 h prior to seeding in 0.3% agar. After two weeks of incubation, colonies were stained with crystal violet and enumerated using Image J. Untreated cells formed multiple colonies, averaging more than 250 colonies per well. Cells treated with rapamycin, however, exhibited impaired tumorigenesis as they failed to form colonies (*p<*0.001) (Figure 6A and B).

### Rapamycin and TMZ treatment synergistically affect ROS production and MMP depolarization

**Figure 7:**
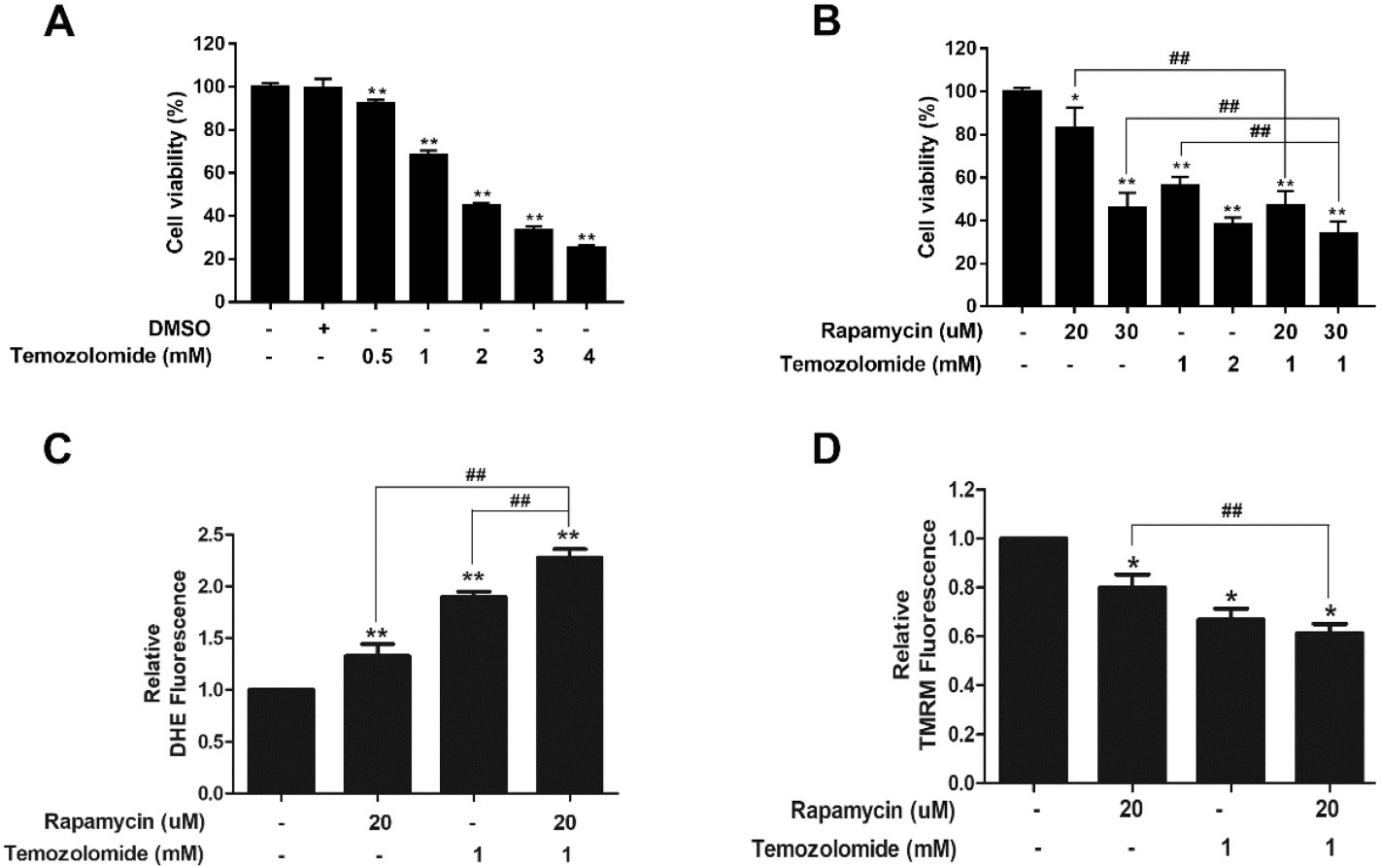
** Rapamycin-TMZ co-treatment enhances mitochondrial dysfunction. A)** TMZ dose response in U87-MG cells. Cells were treated for 24 h with TMZ concentrations from 0.5 to 4 mM **B)** Rapamycin-TMZ co-treatment reduces cell viability. Resazurin viability assay results after incubating cells for 24 h with rapamycin alone, TMZ alone, or rapamycin plus TMZ at the indicated concentrations. C) Co-treatment increases ROS production. Cells were treated as indicated for 24 h with 5 µM DHE added the final 30 min. D) Co-treatment amplifies mitochondrial membrane depolarization. Cells were treated as indicated with 200 nM TMRM added during the final 30 min of the 24 h treatment period. Results are representative of three separate experiments. * *p<*0.05, ** *p<*0.01 and *** *p<*0.001 *vs.* control; ## *p<*0.01.

To assess the clinical relevance of our findings we determined if rapamycin treatment could enhance the cytotoxic effect of TMZ. A viability assay was performed with titrations of TMZ alone (0.5-4 mM) in U87-MG cells. Compared to DMSO controls, TMZ reduced cell viability in a dose-dependent manner (Figure 7A). We chose a concentration of 1 mM TMZ that reduced viability by roughly 40%, for co-treatment studies with rapamycin (Figure 7B). Viability assays showed that TMZ (1 mM) and rapamycin (20 µM) co-treatment therapy significantly reduced viability of cells when compared to 20 µM rapamycin alone (*p<*0.01). Furthermore, when rapamycin was increased to 30 µM, the combination therapy significantly reduced viability compared to TMZ treatment (1 mM) and rapamycin (30 µM) alone (*p<*0.01). The combined effect on mitochondrial health was also observed (Figures 7C and 7D). Figure 7C shows that both rapamycin (20 µM) and TMZ (1 mM) increased ROS production on their own and, when added together, was significantly enhanced (*p<*0.01). In a similar fashion, rapamycin and TMZ both caused depolarization of the mitochondrial membrane (Figure 7D; *p<*0.05). Co-treatment significantly enhanced depolarization when compared to rapamycin treatment alone (*p<*0.01).

## Discussion

The mTOR pathway is elevated in the majority of glioblastomas 5, 6 and inhibiting the pathway has apoptosis-inducing effects on cells 16-18. These effects are observed in glioblastoma with other PI3K/mTOR pathway inhibitors as well, such as a nitric oxide-releasing HIV protease inhibitor, Lopinavir 36. ATP-competitive inhibitors of mTOR, which can block both the mTORC1 and mTORC2 complexes, also exert anti-cancer effects in GBM 37-39. In addition, various rapalogues, such as everolimus and temsirolimus, pan-PI3K inhibitors, dual inhibitors targeting PI3K and mTOR, as well as mTOR and AKT signaling, have yielded promising pre-clinical results within a variety of cancer models 40. However, none of these drugs, along with rapamycin, are currently approved for the treatment of glioblastoma, and only a small handful of these inhibitors are in phase I/II clinical trials. Determining the therapeutic features inherent in treatments that produce a desired response is essential for identifying candidates with a greater chance of being successful in a clinical trial. Our work shows that effects on mitochondrial health should be considered in making this determination.

In our present study, we verified the reported cytotoxic effects of rapamycin in U87-MG cells, showing significant decreases in cell viability, and positive staining of apoptotic markers that were rapamycin dose-dependent (Figure 1). U87-MG cells are known to still be sensitive to both TMZ and rapamycin treatment 41. Our results showed that mTOR signaling is still effectively downregulated, and there is no increase in AKT signaling in response to mTOR inhibition (Figures 2 and 3). Therefore, our results reflect underlying changes that occur in whole cells, and particularly in mitochondria, prior to the development of resistance. We believe this insight into how mitochondria are affected during the initial drug exposure is helpful towards understanding their potential role in developing drug resistance mechanisms. As noted in our introduction, mitochondria are involved in the development of resistance to TMZ 23-25. However, the effect of rapamycin on mitochondria in GBM has received little attention.

Our present study examined the underlying effects on mitochondria during suppression of mTOR signaling and identified mitochondrial parameters amenable to therapeutic targeting. These parameters included disrupting the MMP, increasing ROS production, and altering mitochondrial dynamics. The MMP is the overall charge difference, or electrical gradient, that occurs across the inner mitochondrial membrane (IMM). While some fluctuations in the MMP can be tolerated, sustained irregularities in it can cause various pathologies. As we showed in Figure 4A, there is reduced electrostatic attraction of the cationic dye, TMRM, to the IMM in rapamycin-treated cells, which had a reduced fluorescent signal. This is an indicator that the MMP has become more negative, or depolarized. Under normal physiological conditions, the MMP has a mean value of -140 mV and is able to tolerate naturally occurring fluctuations ranging between -108 and -159 mV 42, 43. Thermodynamic principles dictate that the optimal MMP for maximal ATP production is between -130 to - 140 mV 43. MMP alterations of only 10%, either above or below this optimum range, results in an approximately 90% decrease in ATP synthesis and a roughly 90% increase in the production of harmful ROS 43. MMP depolarization also caused increased ROS production. Consistently, we observed a significant increase in ROS production with rapamycin treatment (Figure 4B). High levels of ROS are not uncommon in hypoxia-prone cells, such as glioblastoma, and undoubtedly, these cells have developed adaptive mechanisms to survive in this stressed environment. Rapamycin treatment appears to increase ROS production beyond U87-MG's tolerance threshold to stimulate apoptosis (Figure 1). Mitochondria, being the prime location of ROS generation, are particularly susceptible to damage given their quick reaction time. Extensive oxidative damage can further disrupt the MMP serving to amplify oxidative stress mechanisms. Alterations in the MMP and in ROS production are the key characteristics of mitochondrial dysfunction 43.

An imbalance in mitochondrial dynamics can also contribute to mitochondrial dysfunction with deadly consequences to the cell. Indeed, the adverse cellular effects have been widely reported in the literature and contribute to a variety of pathological conditions, including cancers, neurodegenerative, cardiovascular, and inflammatory diseases 30, 44. Thus, modulating mitochondrial dynamics is seen as a promising strategy to improve disease outcomes 45. For example, a study by Kriel et al., reported that disruption of mitochondrial bioenergetics, including alterations impairing fission and fusion processes, enhanced apoptosis in glioblastoma cells 46. Upregulation of the fission-promoting protein, DRP1, has been noted in a variety of cancers 30. Typically, cells with increased pDRP1 exhibit more fragmented mitochondria. Conversely, disrupting DPR1 is believed to interfere with quality control by limiting clearance of damaged mitochondria through the process of mitophagy 28. When cells are less able to remove damaged mitochondria, cellular functions become compromised 47. Inhibition of DRP1 in brain tumor initiating cells has been shown to induce cell death and inhibit tumor growth 48. Our own results support these findings. Rapamycin significantly decreased expression of both DRP1 and its helper, FIS1, in a time-dependent manner (Figure 5A and B). This effect may underlie an ability of rapamycin to disrupt what may be an adaptive mechanism of GBM, to increase DRP1/FIS in order to withstand ROS-induced damage to mitochondria that would, under normal circumstances, trigger apoptosis in the affected cells.

Acting in opposition to mitochondrial fission, is mitochondrial fusion, mediated primarily by MFN2 and OPA1. Our results showed that MFN2 is initially upregulated after 4 hours of treatment with rapamycin (Figure 5A). This could likely be in response to cellular stress induced by rapamycin treatment. This increased MFN2 may drive an initial promotion of mitochondrial fusion in an attempt to protect cells from damage. Alternatively, we can consider reports that suggest MFN2 is a tumor suppressor gene and which demonstrate reduced expression of MFN2 in several cancers 49, 50. In this regard, an anti-cancer mechanism has yet to be established, however, it is within the realm of possibilities that fusion, under these circumstances, plays a role beyond the pooling of mitochondrial resources during increased energy demands. Indeed, mitochondrial fusion, mediated by MFN2, has been shown to promote oxidative phosphorylation 51. In this context, increased expression may not have a direct apoptosis-inducing effect, but rather may limit tumor aggression by promoting a return to oxidative phosphorylation within the mitochondria, as opposed to increased glycolytic metabolism typically encountered within glioblastoma cells. The long-term effect of increasing MFN2 expression on tumor transformative potential, absent of a concurrent apoptosis-inducing treatment, was not studied in this present work, but remains an interesting prospect for glioblastoma therapy. That the increase we observed at 4 hours was not sustained after 24 hours may be due to the more overt apoptosis-inducing effects of rapamycin treatment, preventing a further increase in MFN2 expression. Any initial fusion of the OMM, mediated by MFN2, would likely be stalled given the severe depolarization of the MMP across the IMM. Indeed, with this alteration, we also saw a significant, time-dependent decrease in expression of the IMM fusion-promoting protein, OPA1 (Figure 5B). OPA1 has previously been shown to be downregulated when the MMP is disrupted 28, a finding which our results support. In addition to affecting the fusion process, OPA1 has more recently been shown to oppose apoptosis through stabilizing ATP synthases 52. This finding is also in line with our results showing an increase in apoptosis with our loss of OPA1 expression.

mTOR is a master regulator of cell survival and its downregulation by rapamycin has been thought to be key to its apoptosis-inducing effects in glioblastoma. Reduced pS6 (Figure 3C) indicates reduced protein translation/amino acid availability in our glioblastoma cells. This deprivation can lead to significant cellular stress affecting the mitochondria. Not only did rapamycin treatment induce apoptosis directly, but surviving cells experienced significant adverse effects to their mitochondria, namely depolarization of the MMP, increased ROS production, and disruption of mitochondrial dynamics. These surviving cells also exhibited a marked decrease in their ability to form colonies in soft agar - a measure of their transformative potential/tumorigenesis (Figure 6).

Application of these findings could include combining TMZ with mTOR inhibitors, such as rapamycin, or other drugs known to alter mitochondrial function as a new therapeutic strategy. For example, we demonstrated here how adding rapamycin treatment with TMZ increases the anti-cancer effect observed from TMZ treatment alone (Figure 7B). This combination was examined previously by Li et al., in the glioma cell line, U251, and was shown to enhance TMZ-induced autophagic death 53. Unfortunately, the effects on mitochondria were not examined in that study. Our results showed that this co-treatment further enhanced depolarization of the mitochondrial membrane and lead to an increase in the production of toxic ROS (Figures 7C&D), indicating that there is a synergistic anti-cancer effect between these two drugs. Other synergistic effects combining various mTOR inhibitors have been reported in the literature. For example, the combination of nimotuzumab and rapamycin was found to be effective against TMZ-resistant glioma cells 54 and, in an orthotopic xenograft model, a dual mTORC1/2 inhibitor, AZD8055, was shown to inhibit glioblastoma tumor initiating cells synergistically with TMZ 55. Another approach is to combine mTOR and AKT inhibitors in hopes to prevent acquisition of resistance mechanisms mediated by AKT. RES-529, which inhibits both mTORC1/2 and AKT, is a promising candidate in this regard. While its effects on mitochondrial function are not known at this time, RES-529 has received orphan designation by the Food and Drug Administration for the treatment of glioblastoma and prostate cancer 56.

## Conclusions

Despite the development of resistance to conventional GBM therapies such as TMZ, or even in studies utilizing rapamycin for treatment, this study provides proof-of-concept that mitochondria are effective targets for glioblastoma therapy. We show in this study that rapamycin, in addition to exhibiting gross effects on cell morphology, concomitant with the induction of apoptosis, significantly alters mitochondrial health. The overall reduction in mTOR signaling may act as a trigger to amplify cellular stress leading to mitochondrial dysfunction. This lends credence to the concept that we can target mitochondria outside of mTOR signaling or other pathways commonly resulting in unwanted resistance from countermeasures. Taken together, our results indicate that combining rapamycin with TMZ amplified their toxic effect on the mitochondria of glioblastoma cells, and that targeting mitochondria in glioblastoma could be an effective therapeutic approach.

## Abbreviations

AKT: protein kinase B
pAKT: phosphorylated protein kinase B
AV: annexin V
DHE: dihydroethidium
DMEM: dulbecco's modified eagles medium
DMSO: dimethyl sulfoxide
DRP1: dynamin-related protein 1
pDRP1: phosphorylated dynamin-related protein 1
EGFR: epidermal growth factor receptor
ETC: electron transport chain
FIS1: mitochondrial fission 1 protein
GBM: glioblastoma multiforme
IMM: inner mitochondrial membrane
mTOR: mammalian target of rapamycin
p-mTOR: phosphorylated mammalian target of rapamycin
MMP: mitochondrial membrane potential
MOMP: mitochondrial outer membrane permeabilization
MFN1/2: mitofusins 1 and 2
OPA1: optic atrophy 1
p70 S6K: p70 S6 kinase
p-p70 S6K: phosphorylated p70 S6 kinase
PI3K: phosphoinositide 3-kinase
pS6: S6 ribosomal protein
ROS: reactive oxygen species
RTK: receptor tyrosine kinase
TMRM: tetramethylrhodamine, methyl ester, perchlorate
TMZ: temozolomide
